# Cyclosporine Monotherapy in Pediatric Patients With Non-severe Aplastic Anemia: A Retrospective Analysis

**DOI:** 10.3389/fmed.2022.805197

**Published:** 2022-03-07

**Authors:** Hongmin Li, Lingling Fu, Bixi Yang, Hui Chen, Jie Ma, Runhui Wu

**Affiliations:** ^1^Department of Hematology, Beijing Children's Hospital, Capital Medical University, National Center for Children's Health, Beijing, China; ^2^Hematologic Disease Laboratory, Hematology Center, Beijing, China; ^3^Beijing Pediatric Research Institute, Beijing Children's Hospital, Capital Medical University, National Center for Children's Health, Beijing, China

**Keywords:** cyclosporine (CsA), non-severe aplastic anemia (NSAA), children, efficacy, regulatory *T*-cells (Treg cells)

## Abstract

**Objective:**

The management of children with non-severe aplastic anemia (NSAA) is undefined and the efficacies and benefits of immunosuppressive therapy remain inconsistent. The study aimed to investigate the efficacy of Cyclosporine (CsA) monotherapy for pediatric NSAA.

**Methods:**

Clinical data of children with NSAA who had been treated with CsA monotherapy at the outpatient department of Beijing Children's Hospital, Capital Medical University, National Children's Medical Center from January 2017 to March 2021 was collected retrospectively. Patients who had been treated <1 years until the end of follow-up were excluded. Transfusion-independent NSAA was further divided into moderate NSAA and mild NSAA according to the degree of cytopenia. Progression was defined as the development of transfusion-dependent AA or SAA and relapse was considered when treatment failed after initial response.

**Results:**

A total of 95 pediatric patients with NSAA were enrolled in this study with 49 (51.6%) patients confirmed as mild NSAA, 38 (40%) as moderate NSAA and 8 (8.4%) as transfusion-dependent NSAA. The median treatment time of CsA was 22 (12–44) months. The overall response rate (ORR) was 57.9%, with 30.5% CR and 27.4% PR. Unexpectedly, patients with mild NSAA acquired lowest ORR (46.9%), then patients with moderate NSAA (63.2%), while 8 patients who were transfusion-dependent all had an active response to CsA. The granulocyte and megakaryocyte response was 46.9 and 55.8% respectively, while the erythrocyte response rate was as low as 22.5%. Univariate analyses revealed that patients with lower platelet count and higher interleukin 10 level predict an active response to CsA while higher level of fetal hemoglobin (HbF) tended to be a negative factor. Data of Treg cells before and after 1 year's treatment was available in a total number of 40 patients. Paired comparison found that the percentage of Treg cells in CD4+ T cells was decreased after 1 year's treatment of CsA (6.78 ± 2.72 vs. 5.23 ± 2.06, *P* = 0.001),both in responders and non-responders. The degree of decline in Treg cells between two distinctive response groups had no significant difference (*P*>0.05). With a median follow-up time of 22 months, 10.9% of responders relapsed and maintained NSAA while 27.5% of non-responders progressed to SAA or became transfusion-dependent. The overall progression rate was 11.6%.

**Conclusion:**

CsA monotherapy had heterogeneous effects in the treatment of children NSAA Treatment approaches should be hierarchical and individual in clinical. Patients with lower platelet count and higher interleukin 10 level predicted an active response to CsA. While higher level of fetal hemoglobin (HbF) tended to be a negative factor. The percentage of Treg cells in CD4+ T cells was decreased broadly after treatment.

## Introduction

Acquired aplastic anemia (AA) in children is a rare, life-threatening disorder characterized by pancytopenia and hypocellular bone marrow. The degree or severity of acquired AA is defined by peripheral blood cell counts in the presence of a hypocellular bone marrow (1, 2). For children with severe AA (SAA), bone marrow transplantation from a matched related donor (MRD) and immunosuppressive therapy (IST) using antithymocyte globulin (ATG) and cyclosporine (CsA) for those who lack a MRD have been considered as the first-line therapy, with an overall long-term survival rate of 90% (3–5). However, for children with non-severe AA (NSAA), there is no standard or widely effective treatment approaches. The British Committee for Standards in Hematology (BCSH) recommends only supportive care or non-treatment follow-up in transfusion-independent NSAA patients (6). Actually, variety of early interventions were given to children with NSAA in the real word, according to the study which reported a high rate of progression to SAA when treated with supportive care alone (7). CsA monotherapy or combined with ATG, ATG only, eltrombopag, androgens, traditional Chinese medicine, etc. are common treatment options clinically (8–12). The efficacies and benefits of this approaches remains inconsistent, partly because the large heterogeneity of the disease and very few clinical trials have been conducted.

The present study reported the efficacy of CsA monotherapy in the treatment of NSAA based on a pediatric cohort with certain number of samples retrospectively. Factors which may be associated with the efficacy were analyzed in order to identify patients who can benefit from the treatment and provide data support for stratified treatment of NSAA.

## Materials and Methods

### Patients

Clinical data of children with NSAA who had been treated with CsA monotherapy at the outpatient department of Beijing Children's Hospital, Capital Medical University, National Children's Medical Center from January 2017 to March 2021 was collected retrospectively. Patients who had been treated <1 year until the end of follow-up were excluded. Bone marrow biopsy, chromosome karyotype analysis and next generation sequencing were performed on all patients. The diagnosis of acquired AA was considered for patients without dysplastic hematopoietic cells, abnormal karyotype, and lacking classic congenital anomalies such as *FANC, SBDS, DKC1* (1). The definition of severity of AA was based on the Camitta criteria (13). Furthermore, transfusion-independent NSAA was divided into two groups according to a previous study (14): 1) Moderate NSAA had to meet at least 2 of the following criteria: reticulocyte count <60 × 10^9^/L, platelet count <50 × 10^9^/L, neutrophil count <1.0 × 10^9^/L and not meeting the criteria for SAA. 2) Mild NSAA was defined as transfusion-independent NSAA that did not meet the criteria of Moderate NSAA. A positive PNH clone was defined as the percentage of CD235a+CD55-/CD235a+CD59- red blood cells ≥ 1% or the percentage of CD24-/Flear- granulocytes ≥ 1% (per 10,000 nucleated cells) by flow cytometry. The study was approved by the ethnic committee of Beijing Children's Hospital, Capital Medical University, National Children's Medical Center.

### Treatments

Cyclosporine was administered orally at an initial dose of 3–5 mg/kg per day. The dose was adjusted according to plasma CsA concentration to maintain a trough plasma concentration of 150–200 ng/ml. Then the oral dose was decreased gradually 1 year after the blood cells reached a plateau. The total course of treatment was 3–4 years.

### Responses

Refer to the 2016 British Hematology Guidelines (6), a complete response (CR) was defined as hemoglobin normal for age and gender, absolute neutrophil count >1.5 × 10^9^/L and platelet count >100 × 10^9^/L; a partial response (PR) was defined as transfusion independence (if previously dependent) or at least one cell line response: (1) Erythrocyte response defined as hemoglobin concentration close to normal or increase of baseline >30 g/L (if initially <60 g/L) 0.2) Granulocyte response defined as neutrophils close to normal or increase of baseline >0.5 × 10^9^/L (if initially <0.5) 0.3. Megakaryocyte response defined as platelets increase of baseline >20 × 10^9^/L (if initially <20) or doubling of baseline (if initially ≥20) or close to normal. No response (NR) was defined as blood counts worse, or do not meet criteria above. Patients who acquired PR or CR were considered responsive. We defined progression as the development of transfusion-dependent AA or SAA and relapse as treatment failure after initial response.

### Regulatory *T*-Cells

Multicolor flow cytometry for detection of CD4+CD25+foxP3+ regulatory *T*-cells (Treg cells) was performed on blood samples of patients at the time of initial treatment and 1 year after treatment.

### Statistical Analysis

The SPSS 22.0 software was used for data processing and statistical analysis. The normal distribution of the variables was examined by the Kolmogorov-Smirnov test and data distributed normally was presented as means with standard deviation. Comparison between two different efficacy groups were analyzed by unpaired *t*-test or non-parametric Mann–Whitney U tests for continuous variables. Pearson's chi-square test was used for counting variables. Binary logistic regression was used for multivariate analysis. Comparison of Treg cells before and after treatment was conducted by paired *T*-test. Kaplan Meier curves were used for the analysis of progression-free survival. *P* < 0.05 was considered as statistically significant.

## Results

### Patient Characteristics

A total of 95 pediatric patients with NSAA were enrolled in this study, including 48 (50.5%) boys, and 47 (49.5%) girls, with a median age of 7 (1–14) years. Among them, 49 (51.6%) patients were confirmed as mild NSAA,38 (40%) patients confirmed as moderate NSAA and 8 (8.4%) patients confirmed as transfusion-dependent NSAA.54 (56.8%) patients had a positive PNH clone. The median course of disease before CsA treatment was 7 (1–132) months, and 32.63% children had been treated with different drugs such as glucocorticoid, androgens, traditional Chinese medicines, with no effects. All patients were treated with CsA only, and the median treatment time was 22 (12–44) months. None of the patients stopped the drug by the end of the follow-up. The baseline characteristics of the patients were shown in Table 1.

**Table 1:** Baseline characteristics of pediatric NSAA patients.

**Patient characteristics**	**Values**
Sex, *n* (%)	
Male	48 (50.5)
Female	47 (49.5)
Median age, y	7 (1–14)
Neut (median, × 10^9^/L)	1.19 (0.21–5.43)
HGB (median, g/L)	106 (14–140)
PLT (median, × 10^9^/L)	33 (2–88)
Positive PNH clone, *n* (%)	54 (56.8)
Severity, *n* (%)	
Mild	49 (51.6)
Moderate	38 (40)
Transfusion-dependent	8 (8.4)
Median course of disease, m	7 (1–132)
Previous treatments, *n* (%)	
Glucocorticoid	16 (16.8)
Androgen	1 (1.1)
Traditional Chinese medicines	11 (1.6)
Others^*^	3 (3.2)
Median time of CsA treatment, m	22 (12–44)

* *Includes levamisole, rituximab, and intravenous immunoglobulin*.

*Neut, neutrophil; HGB, hemoglobin; PLT, platelet; y, years; m, months*.

### Responses

**Figure 1:**
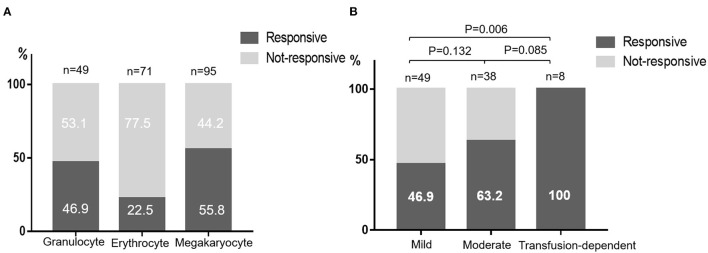
Responses to CsA monotherapy. **(A)** Responses of different blood cell lines. **(B)** Responses of different NSAA groups classified according to the degree of cytopenia.

After a median time of 22 months' treatment,30.5% (29/95) patients met the criterion of CR, and 27.4% (26/95) met the criterion of PR. The overall response rate (ORR) was 57.9%. The median duration from treatment to initial response was 2.5 (0.5–12) months and the median time to complete response was 8 (1.5–24) months. Anemia was present in 71 patients at the initial of treatment and the erythrocyte response rate was 22.5%. The granulocyte and megakaryocyte response was 46.9 and 55.8% respectively (Figure 1A). For patients diagnosed with mild NSAA, 15 (30.6%) acquired a response of CR and 8 (16.3%) acquired a response of PR. The rate of CR and PR of patients with moderate NSAA was 28.9 and 34.2% respectively. Unexpectedly, 8 patients who were transfusion-dependent all had an active response to CsA, with 3 CR and 5 PR (Figure 1B).

### Efficacy Related Factors

Factors which may be associated with the efficacy of CsA monotherapy were further analyzed. Univariate analyses revealed that lower platelet count and higher interleukin 10 (IL-10) level in NSAA patients predict an active response to CsA. Patients with high level of fetal hemoglobin (HbF) tended to have negative response to CsA (Table 2). Positive PNH clone did not make patients more effective to CsA. However, none independent influencing factor was found in multivariate analysis (Table 3).

**Table 2:** Univariate analyses of efficacy related factors.

	**Responders (*n* = 55)**	**Non-responders (*n* = 40)**	***P***
Sex, *n* (%)			0.358
Male	30 (54.5)	18 (45)	
Female	25 (45.5)	22 (55)	
Median age, y	7 (1–14)	7 (2–14)	0.605
Neut (median, × 10^9^/L)	1.12 (0.27–5.43)	1.2 (0.21–3.66)	0.778
HGB (median, g/L)	106 (14–140)	105 (32–133)	0.987
PLT (median, × 10^9^/L)	30 (2–88)	44 (14–82)	**0.031**
HBF (%)	5.87 (0–37.3)	10 (0–23.37)	**0.02**
Treg cells before treatment(Mean ± SD, %)	6.64 ± 2.43	7.0 ± 2.32	0.547
Positive PNH clone, *n* (%)	33 (60)	21 (52.5)	0.466
IFN	0.2 (0–33.27)	0 (0–20.3)	0.369
TNF	2.36 (0–92.58)	3.05 (0–179.7)	0.464
IL-10	2.21 (0–178)	1.42 (0–10.51	**0.033**
IL-6	8.76 (0–2,787)	26.93 (0–774)	0.146
IL-4	0 (0–0.26)	0 (0–3.13)	0.492
IL-2	0 (0–2.27)	0 (0–20)	0.478
Median course of disease, m	6.25 (1–108)	10.5 (1–132)	0.311
Median time of CsA treatment, m	24 (12–44)	19 (12–38)	0.074

*Neut, neutrophil; HGB, hemoglobin; PLT, platelet; y, years; m, months; HBF, fetal hemoglobin; Treg cells, regulatory T-cells; IFN, Interferon; IL-10/6/4/2, Interleukin 10/6/4/2. P values less than 0.05 was bolded*.

**Table 3:** Multivariate analyses of efficacy related factors by Binary logistic regression.

	**B**	**S.E**.	***P***	**Exp (B)**	**95% EXP (B)**
PLT	−0.023	0.012	0.050	0.977	0.955–1.000
HBF	−0.055	0.033	0.096	0.946	0.886–1.010
IL10	0.055	0.075	0.465	1.056	0.912–1.224
Constant	1.581	0.636	0.013	4.858	

*PLT, platelet; HBF, fetal hemoglobin; IL-6 Interleukin 6*.

### Regulatory *T*-Cells

**Figure 2:**
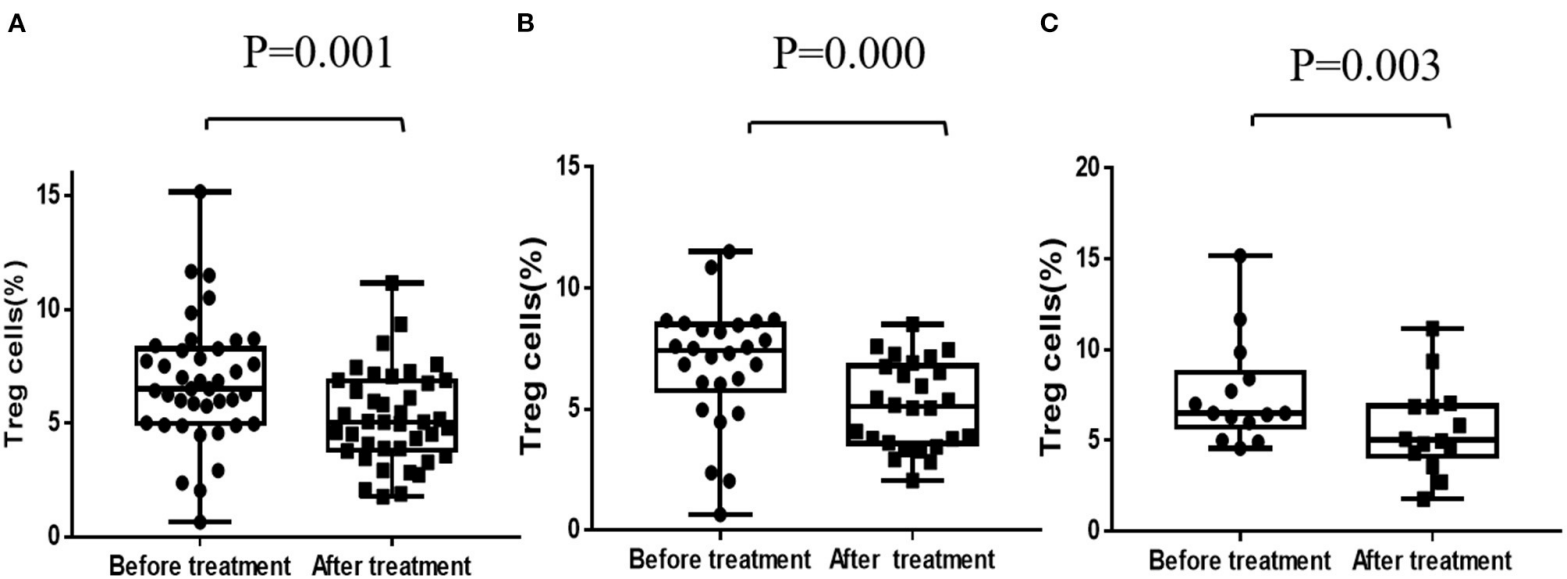
Regulatory *T*-cells before and after CsA treatment. **(A)** Regulatory *T*-cells of all patients (*n* = 40). **(B)** Regulatory *T*-cells of responders (*n* = 26). **(C)** Regulatory *T*-cells of non-responders (*n* = 14).

Data of Treg cells before and after 1 year's treatment was available in a total number of 40 patients. Paired comparison found that the percentage of Treg cells in CD4+ *T*-cells was decreased after 1 year's treatment of CsA (6.78 ± 2.72 vs. 5.23 ± 2.06, *P* = 0.001), both in responders and non-responders. The degree of decline in Treg cells between two distinctive response groups had no significant difference (*P* < 0.05) (Figure 2).

### Follow-Up and Outcomes

**Figure 3:**
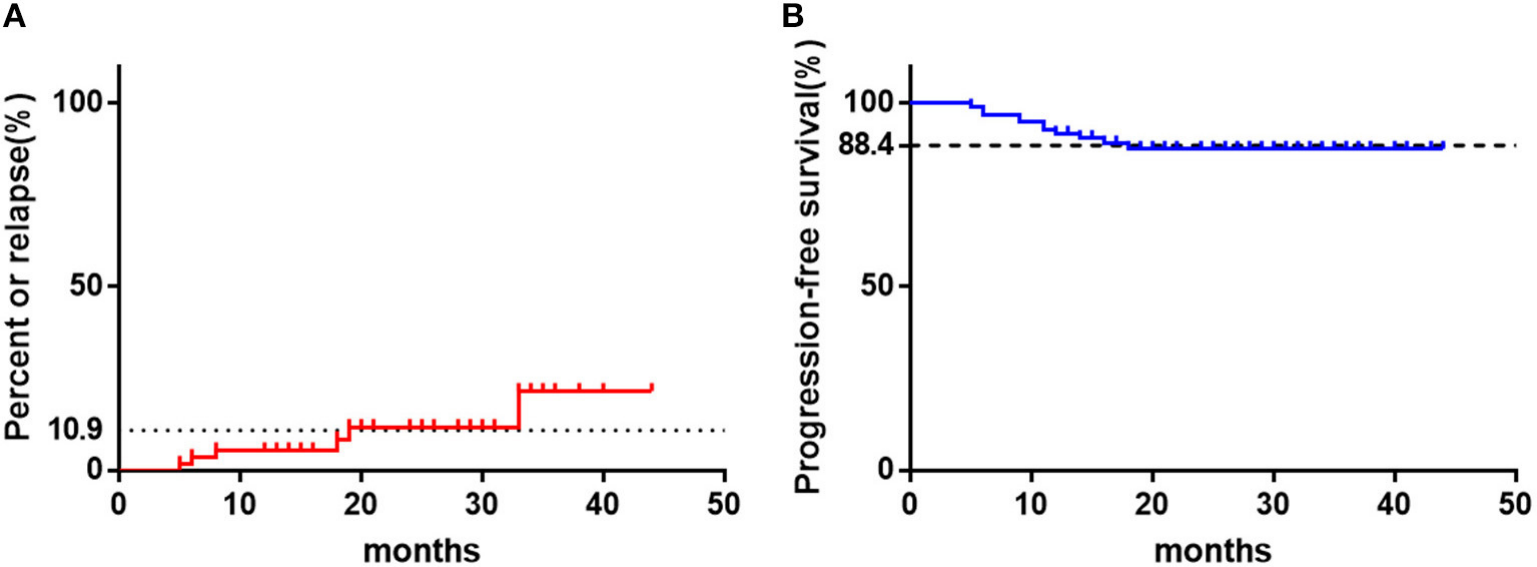
The clinical outcomes of children with NSAA treated with CsA with a median follow-up time of 22 months. **(A)** The relapse percent of patients in responders (*n* = 55). **(B)** The rate of progression-free survival of all patients as time. The horizontal dotted line represents the final values.

With a median follow-up time of 22 months, 10.9% of the responders relapsed and maintain NSAA, without further progression. The median time from initial response to relapse was 13 (5–33) months. However, 11 patients who did not responsive to CsA monotherapy progressed to SAA or became transfusion-dependent.The overall progression rate was 11.6% and the median time from CsA treatment to progression was 11 (5.5–18) months (Figure 3).

## Discussion

Very few clinical trials have been conducted for patients, including adults, with NSAA and when treatment for NSAA patients should begin is controversial. The British guideline for treatment of AA recommend that patients should only be observed, or treated with supportive care, until they become transfusion-dependent (6). However, according to the study of St. Jude Children's Research Hospital, at a median follow-up of 66 months, 2/3 of pediatric moderate NSAA patients progressed to SAA (7). Similarly, another Asian medical center also reported high incidence of disease progression for NSAA patients without definitive treatment, with progression-free survival 62% at 60 months and 22% at 120 months after diagnosis (10). However, for patients treated with CsA combined with stanozolol, only 9.2 % NSAA patients progressed to SAA at 26.5 months after diagnosis (15). In the current study, the overall progression rate was 11.6%, with a median follow-up time of 22 months. None of patients who had an active response to CsA progressed though 10.9% of them relapsed. While 27.9% of patients who failed to response to CsA progressed to a more severe stage. Above data indicated that effective treatment seems to be able to decrease the rate of disease progression in children with NSAA. Thus, identify the beneficiaries early and give them appropriate treatment is essential.

In view of the satisfying outcomes of immunosuppressive therapy (IST) in the treatment of SAA, several studies in NSAA have reported variable responses to kinds of IST regimes. A prospective randomized multicenter study concluded that the combination of ATG and CSA is superior to CSA alone for transfusion-dependent NSAA patients, with the ORR of 74 and 46% at 6 months respectively (16). Wang and colleagues treated NSAA patients with CsA and stanozolol and 19.6% patients showed a complete remission (15). In our study, the responses of patients to CsA were heterogeneous, with the ORR 46.9% in mild NSAA, 63.2% in moderate NSAA and 100% in transfusion-dependent NSAA. We think this may be related to the complicated pathogenesis of AA. The most favored model is that of a dysregulated immune system leading to autoreactive *T-cell* destruction of hematopoietic stem and progenitor cells. This is well reflected in SAA patients with expanded CD8+ *T*-cells and increased TNFα and INFγ levels (17–19). However, the same immunological abnormality is rarely found in NSAA. To these patients, other mechanisms such as telomere abnormalities, genetic factors and clonal hematopoiesis may also be involved (20). Especially for children acquired NSAA, which is difficult to distinguish with inherited bone marrow failure syndrome and refractory cytopenia (21). Therefore, the author thinks that to mild NSAA patients, supportive care or only observation until disease progressed was suitable since no effective treatment was available. The ORR of patients with transfusion-dependent was as high as 100%. One possible reason was that transfusion-dependent patients were more likely to be lost to follow-up if the drug did not work quickly. Another reason was that 6 of 8 transfusion-dependent patients in our study were platelet transfusion-dependent and further univariate analyses revealed that lower platelet count predicted an active response to CsA. Meanwhile, patients with higher IL-10 level seem to have a better response to CsA, which is easy to be understood since IL-10 are defined as stimulator of hematopoiesis (22). Higher level of HbF tend to have negative response to CsA monotherapy. It may be an indicator of clonal hematopoiesis as previous study have reported a marked increase in the number of F blasts in the bone marrow of patients with myelodysplastic syndromes (MDS) (23). However, none independent influencing factor was found in multivariate analysis. We believed that on the one hand, the disease itself had great heterogeneity with complex and diverse pathological mechanism, which make it difficult to be affected by an independent factor. On the other hand, limited by retrospective research, patients included were heterogeneous too, with different medical histories and previous treatments. Thus, long-term prospective follow-up studies are necessary. In the current study, erythrocyte response rate was much lower than that of granulocyte and megakaryocyte. Thus, for NSAA patients suffering from anemia, more aggressive treatment such as CsA combined with ATG, androgen or eltrombopag should be considered (24, 25).

Treg cells are important in regulating the differentiation of *T*-cells and inhibiting the toxicity of activated CD8+*T*-cells, and thus play a crucial role in maintaining normal function of the *T*-cells (26). It is reported that Treg cells, as marked by CD4+CD25+ and Foxp3, is obviously lower and functional impaired in AA patients and Treg cells injection can increase residual bone marrow cells and peripheral blood cells of AA mice (27, 28). In our study, however, the percentage of Treg cells in CD4+ *T*-cells was decreased after 1 year's treatment of CsA, both in responders and non-responders. Similarly, previous studies had reported that CsA significantly impaired the function of CD4+CD25+ Treg cells by inducing interleukin-2 (IL-2) and interferon-gamma (IFN-gamma) secretion (29). But whether this will lead to patients' impaired immune tolerance is unknown. Rapamycin, another effective immunosuppressant, does not inhibit the function of CD4 (+)CD25 (+) Treg cells (30). Futhermore, rapamycin, but not CsA, reduced the proportion of memory and effector *T*-cells and maintained a pool of naïve *T*-cells in murine models of immune-mediated bone marrow failure (31). This implies that rapamycin or other effective immunosuppressant could be used as alternative options in the treatment of aplastic anemia. But randomized controlled studies based on large samples and long-term follow-up are necessary.

In conclusion, pediatric NSAA is highly complicated and heterogeneous. Treatment approaches should be hierarchical and individual. For patients with moderate or transfusion-dependent NSAA, CsA monotherapy had certain effects. For patients suffering from anemia, more aggressive treatment should be considered. For patients with mild NSAA, CsA is not recommended and a prospective randomized trial of early intervention with IST or observation alone until disease progression is urgently needed. The percentage of Treg cells were decreased by CsA, but whether this affects patients' long-term outcomes needs to be further studied.

## Data Availability Statement

The original contributions presented in the study are included in the article/supplementary material, further inquiries can be directed to the corresponding author/s.

## Ethics Statement

The studies involving human participants were reviewed and approved by the Ethics Committee of Beijing Children's Hospital, Capital Medical University, National Children's Medical Center. Written informed consent to participate in this study was provided by the participants' legal guardian/next of kin.

## Author Contributions

JM and HL designed the study and wrote the manuscript. HL, LF, BY, and HC collected the medical records and conducted data processing. RW reviewed and corrected this manuscript professionally. All authors reviewed the manuscript and approved the final submission of the manuscript.

## Funding

This work was supported in part by grants from the National Natural Science Foundation of China (No. 81970111), Beijing Natural Science Foundation of China (No. 7192064), the Pediatric Medical Coordinated Development Center of Beijing Municipal Administration of Hospitals (No. XTZD20180205), and National Science and Technology Key Projects (No. 2017ZX09304029001).

## Conflict of Interest

The authors declare that the research was conducted in the absence of any commercial or financial relationships that could be construed as a potential conflict of interest.

## Publisher's Note

All claims expressed in this article are solely those of the authors and do not necessarily represent those of their affiliated organizations, or those of the publisher, the editors and the reviewers. Any product that may be evaluated in this article, or claim that may be made by its manufacturer, is not guaranteed or endorsed by the publisher.

## References

[1] Hartung HD Olson TS Bessler M. Acquired aplastic anemia in children. Pediatr Clin North Am. (2013) 60:1311–36. 10.1016/j.pcl.2013.08.011 24237973 PMC3894991

[2] Yoshida N Kojima S. Updated guidelines for the treatment of acquired aplastic anemia in children. Curr Oncol Rep. (2018) 20:67. 10.1007/s11912-018-0716-8 29961134

[3] Yoshida N Kobayashi R Yabe H Kosaka Y Yagasaki H Watanabe K. First-line treatment for severe aplastic anemia in children: bone marrow transplantation from a matched family donor versus immunosuppressive therapy. Haematologica. (2014) 99:1784–91. 10.3324/haematol.2014.109355 25193958 PMC4258757

[4] Dufour C Pillon M Socie G Rovo A Carraro E Bacigalupo A. Outcome of aplastic anemia in children. a study by the severe aplastic anemia and pediatric disease working parties of the European group blood and bone marrow transplant. Br J Haematol. (2015) 169:565–73. 10.1111/bjh.13297 25683884

[5] Jeong DC Chung NG Cho B Zou Y Ruan M Takahashi Y. Long-term outcome after immunosuppressive therapy with horse or rabbit antithymocyte globulin and cyclosporine for severe aplastic anemia in children. Haematologica. (2014) 99:664–71. 10.3324/haematol.2013.089268 24213150 PMC3971076

[6] so Bown N Cavenagh J Dokal I Foukaneli T Hill A. Guidelines for the diagnosis and management of adult aplastic anemia. Br J Haematol. (2016) 172:187–207. 10.1111/bjh.13853 26568159

[7] Howard SC Naidu PE Hu XJ Jeng MR Rodriguez-Galindo C Rieman MD. Natural history of moderate aplastic anemia in children. Pediatr Blood Cancer. (2004) 43:545–51. 10.1002/pbc.20131 15382271

[8] Matsuda K Koya J Arai S Nakazaki K Nakamura F Kurokawa M. Cyclosporine therapy in patients with transfusion-independent non-severe aplastic anemia: a retrospective analysis. Intern Med. (2019) 58:355–60. 10.2169/internalmedicine.1372-18 30146592 PMC6395135

[9] Geng W Kearney S Nelson S. Upfront eltrombopag monotherapy induces stable hematologic remission in pediatric patients with nonsevere idiopathic aplastic anemia. Pediatr Blood Cancer. (2018) 65:e27290. 10.1002/pbc.27290 29932285

[10] Jiang S Wang Y Shi W Shao Y Qiao X Lin J. The benefit of ATG in immunosuppressive therapy of children with moderate aplastic anemia. Pediatr Hematol Oncol. (2009) 26:313–20. 10.1080/08880010902771549 19579077

[11] Wang SC Li YS Chen XJ Zou Y Yang WY Liu TF. [114 children with acquired non-severe aplastic anemia benefitted from androgen]. Zhongguo Shi Yan Xue Ye Xue Za Zhi. (2011) 19:793–7. 21729573

[12] Zhu N Wu D Ye B. The progress of traditional chinese medicine in the treatment of aplastic anemia. J Transl Int Med. (2018) 6:159–64. 10.2478/jtim-2018-0031 30637201 PMC6326026

[13] Camitta BM. Criteria for severe aplastic anemia. Lancet. (1988) 1:303–4. 10.1016/S0140-6736(88)90388-1 2893118

[14] Hama A Takahashi Y Muramatsu H Ito M Narita A Kosaka Y. Comparison of long-term outcomes between children with aplastic anemia and refractory cytopenia of childhood who received immunosuppressive therapy with antithymocyte globulin and cyclosporine. Haematologica. (2015) 100:1426–33. 10.3324/haematol.2015.128553 26273061 PMC4825303

[15] Wang S Chen Y Zou Y Zheng Y Zhu X. The progression risk factors of children with transfusion-independent non-severe aplastic anemia. Int J Hematol. (2013) 97:210–5. 10.1007/s12185-013-1263-6 23361447

[16] Marsh J Schrezenmeier H Marin P Ilhan O Ljungman P McCann S. Prospective randomized multicenter study comparing cyclosporin alone versus the combination of antithymocyte globulin and cyclosporin for treatment of patients with nonsevere aplastic anemia: a report from the European blood and marrow transplant (ebmt) severe aplastic anemia working party. Blood. (1999) 93:2191–5. 10.1182/blood.V93.7.2191.407a03_2191_2195 10090926

[17] Fang J Lin L Wang Y Lin D Liu C Sunlong Q. Regulatory *T*-cells and CD20 (+) B cells in pediatric very severe aplastic anemia: possible clinical markers for evaluating the therapeutic efficacy and prognosis. Hematology. (2018) 23:823–7. 10.1080/10245332.2018.1498566 29996743

[18] Kordasti S Marsh J Al-Khan S Jiang J Smith A Mohamedali A. Functional characterization of CD4+ *T*-cells in aplastic anemia. Blood. (2012) 119:2033–43. 10.1182/blood-2011-08-368308 22138514

[19] Sloand E Kim S Maciejewski JP Tisdale J Follmann D Young NS. Intracellular interferon-gamma in circulating and marrow *T*-cells detected by flow cytometry and the response to immunosuppressive therapy in patients with aplastic anemia. Blood. (2002) 100:1185–91. 10.1182/blood-2002-01-0035 12149196

[20] Schoettler ML Nathan DG. The pathophysiology of acquired aplastic anemia: current concepts revisited. Hematol Oncol Clin North Am. (2018) 32:581–94. 10.1016/j.hoc.2018.03.001 30047412 PMC6538304

[21] Iwafuchi H. The histopathology of bone marrow failure in children. J Clin Exp Hematop. (2018) 58:68–86. 10.3960/jslrt.18018 29998978 PMC6413145

[22] Gu L Fu B Sui X Xu H. Abnormal expression of b10 cell frequencies: possible relation to pathogenesis and disease severity of aplastic anemia. Rev Assoc Med Bras. (1992) (2019) 65 (5):637-46. 10.1590/1806-9282.65.5.637 31166440

[23] Choi JW Kim Y Fujino M Ito M. Significance of fetal hemoglobin-containing erythroblasts (F blasts) and the F blast/f cell ratio in myelodysplastic syndromes. Leukemia. (2002) 16:1478–83. 10.1038/sj.leu.2402536 12145688

[24] Imada K Obara N Iida H Imajo K Maeda T Usuki K. Eltrombopag in combination with rabbit anti-thymocyte globulin/cyclosporine a in immunosuppressive therapy-naive patients with aplastic anemia in Japan. Intern Med. (2021) 60:1159–68. 10.2169/internalmedicine.6063-20 33229810 PMC8112980

[25] Jalaeikhoo H Khajeh-Mehrizi A. Immunosuppressive therapy in patients with aplastic anemia: a single-center retrospective study. Plos One. (2015) 10:e126925. 10.1371/journal.pone.0126925 25970182 PMC4430492

[26] Miyara M Yoshioka Y Kitoh A Shima T Wing K Niwa A. Functional delineation and differentiation dynamics of human CD4+ *T*-cells expressing the FoxP3 transcription factor. Immunity. (2009) 30:899–911. 10.1016/j.immuni.2009.03.019 19464196

[27] Shi J Ge M Lu S Li X Shao Y Huang J. Intrinsic impairment of CD4 (+)CD25 (+) regulatory *T*-cells in acquired aplastic anemia. Blood. (2012) 120:1624–32. 10.1182/blood-2011-11-390708 22797698

[28] Chen J Ellison FM Eckhaus MA Smith AL Keyvanfar K Calado RT. Minor antigen h60-mediated aplastic anemia is ameliorated by immunosuppression and the infusion of regulatory *T*-cells. J Immunol. (2007) 178:4159–68. 10.4049/jimmunol.178.7.4159 17371972

[29] Miroux C Morales O Carpentier A Dharancy S Conti F Boleslowski E. Inhibitory effects of cyclosporine on human regulatory *T*-cells *in vitro*. Transplant Proc. (2009) 41:3371–4. 10.1016/j.transproceed.2009.08.043 19857752

[30] Bocian K Borysowski J Wierzbicki P Wyzgal J Klosowska D Bialoszewska A. Rapamycin, unlike cyclosporine A, enhances suppressive functions of in vitro-induced CD4+CD25+ Tregs. Nephrol Dial Transplant. (2010) 25:710–7. 10.1093/ndt/gfp586 19903662

[31] Feng X Lin Z Sun W Hollinger MK Desierto MJ Keyvanfar K. Rapamycin is highly effective in murine models of immune-mediated bone marrow failure. Haematologica. (2017) 102:1691–703. 10.3324/haematol.2017.163675 28729300 PMC5622853

